# Man *versus *the Microbe

**Published:** 1905-03-04

**Authors:** 


### MAN *VERSUS* THE MICROBE.

Last Saturday, at a meeting of the Health Society, over which Professor MacFadyen presided, Sir Wm. J. Collins delivered a lecture entitled Man *versus* The Microbe, in which he pointed out that the question of public health is not for the medical man alone, but for every man and woman to consider and study. Neither Chadwick nor Pasteur were medical men. On the other hand, since the public boards began to consider the health as well as the wealth of the nations, it has become the duty of the medical man to take the place on such boards as his education fits him to fill. During the late Queen's reign great advances were made in public health by the municipal governments. Chadwick and other leaders did much to bring about these reforms, but they were helped by the trend of public thought. At the same time, the scientists who by means of the microscope had learnt much about the minute forms of life began to learn about the bacilli and micrococci. At last it seems that the bacillus is to overshadow man himself, for the scientist, with his eye glued to his microscope is apt to lose sight of man and only look at the micro-organism as the cause of disease, although Virchow and other teachers in the infancy of bacteriology showed that there must be a predisposition before the micro-organism can take root. There are two classes, Sir William remarked, working for the health of the public. The first studies the environment of man as the cause of disease, whilst the second studies the micro-organisms which are responsible and tries to render man immune to these. Some have thought that these two classes are opposed, but we should try and combine them, ever remembering that the vigour of man's own life must be our first aim. Sir William holds that the germ theory of disease has been carried to an extreme. In the case of consumption, for example, it may be questioned whether the scientist or the bacteriologist has been the more reliable. Professor Koch taught that consumption could be cured whilst Sir Thomas Barlow says that we must speak with bated breath on its cure, we must aim at its arrest. Probably more is being done by segregation and the improvement of surroundings than by bacteriology, as is shown by the methods in which certain diseases have been stamped out from amongst the lower animals. With regard to bacteriology Sir William believes that Darwin's evolution theory should be put into practice, and points out, in favour of this opinion, how one animal is subject to certain diseases to which others are immune, how the diphtheria baccillus will be found in one person's throat producing no ill results, whilst in another it produces symptoms of this disease. In conclusion, he remarks that healthy blood is the best germicide, and that sanitation has not been pushed off the stage by bacteriology, but still holds its position.

